# Impact of replacing sow milk with milk replacer on growth performance, intestinal development, bacterial profile and muscular maturation in neonatal and nursery piglets

**DOI:** 10.3389/fvets.2025.1565039

**Published:** 2025-04-14

**Authors:** Yuwei Zhang, Shiya Liu, Qiang Zhou, Yang Liu, Liang Hu, Ruinan Zhang, Zhengfeng Fang, Yan Lin, Shengyu Xu, Bin Feng, Yong Zhuo, De Wu, Lianqiang Che

**Affiliations:** ^1^Key Laboratory of Animal Disease-Resistant Nutrition of Sichuan Province, Institute of Animal Nutrition, Sichuan Agricultural University, Chengdu, China; ^2^College of Food Science, Sichuan Agricultural University, Ya’an, China

**Keywords:** swine, milk formula, intestinal health, body composition, microbiome

## Abstract

Along with the increasing litter sizes in pig industry, using milk replacer (MR) as a nutrient supplement has been widely practiced, yet the effects of replacing sow milk (SM) with MR on growth and development of piglets remain unclear. This study evaluated the differential effects of MR versus SM on growth performance, body composition, muscle fiber types, and intestinal health of piglets during the neonatal and nursery periods. Forty 2-day-old piglets, selected from 10 healthy sows, were randomly divided into two groups receiving either SM or MR ad libitum until postnatal day 23 (PND 23), then transitioned to be fed with nursery diet until PND 37. Blood, muscle, and intestinal tissues, along with colonic digesta and carcass samples, were collected on PND 12 (*n* = 10) and PND 37 (*n* = 10) for analysis of parameters related to intestinal function, microbiota composition and muscular development. The results showed that MR-fed piglets had lower average daily gain (ADG) and higher diarrhea index during the neonatal period. During the nursery period, however, MR-fed piglets had significantly higher average daily feed intake (ADFI) and ADG. Compared to SM-fed piglets, MR-fed piglets had a lower percentage of fast twitch fibers, but a higher percentage of slow twitch fibers on PND 12, along with lower body fat content on both PND 12 and PND 37. In addition, MR-fed piglets had significantly deeper crypt depth, increased mRNA expressions of inflammatory genes, and lower alpha diversity on PND 12. On PND 37, however, MR-fed piglets had higher villus height, increased sucrase activity and alpha diversity. On PND 12, likewise, MR-fed piglets were enriched with *Prevotella* associated with diarrhea, while SM-fed piglets were enriched with *Lachnospiraceae* associated with body fat deposition. In contrast, on PND 37, MR-fed piglets were enriched with commonly recognized beneficial bacteria, such as f_Muribaculaceae, *g_Prevotellaceae_NK3B31_group*, f_Oscillospiraceae and f_Rikenellaceae. These findings indicate that piglets fed MR experienced temporary growth check and intestinal complications in neonatal period, but intriguingly MR piglets had higher feed intake, compensatory growth, and recovery of intestinal function during the nursery period.

## 1 Introduction

Sows have been selected for larger litter sizes, but their nursing capacity has not kept pace (1, 2), resulting in more piglets than they can care for, which lowers weaning weights and increases mortality (3, 4). Providing milk replacer (MR) to neonatal piglets is a strategy to ensure adequate nutrition intake and piglets growth (5). It has been found that piglets fed MR had higher weaning weights than those fed sow milk (SM), with this weight advantage being maintained throughout the nursery period (6). However, other studies reported short-term or persistent growth check in MR-fed piglets, indicating that the effects of MR on growth and healthy of neonatal and nursery piglet still needs further investigation (7, 8).

The body composition of pigs is closely linked to their nutrient intake. The nutritional value and quality of MR are generally inferior to SM, especially in fat content (9), which is crucial for the survival of neonatal piglets. Previous studies indicate that piglets fed MR from 4 days of age have lower body fat compared to those fed SM (10, 11). However, it remains to be investigated whether the impact of MR feeding on piglet body composition extends into the nursery period. Additionally, muscle maturation depends on nutrition, as muscle fiber types can transform despite the number of fibers being predetermined before birth (12). The critical period for muscle fiber type transformation occurs from birth to weaning, during which oxidative fibers decrease and glycolytic fibers increase sharply, especially in the first 2 weeks (13–15). However, little is known about the impact of replacing SM with MR on this fiber type transformation during the neonatal period.

Early life nutritional status is crucial for intestinal development and microbiota composition. Research shows that bioactive substances in SM support piglet intestinal development and maturation (16). It has been observed piglets fed MR experience a higher diarrhea incidence compared to those fed SM (7), likely due to the poor adaptive immunity and impaired intestinal function, resulting from the absence of bioactive substances in MR (17, 18). Moreover, the gut microbiome appears to be differentially regulated in piglets fed MR relative to SM (19, 20) and the effects of early nutrition on the gut microbiome may program to affect the post-weaning bacterial colonization and related gut function (21, 22).

In this study, therefore, we aimed to investigate the effects of replacing SM with MR on growth performance, body composition, intestinal development and muscular maturation of piglets during neonatal and nursery period.

## 2 Materials and methods

### 2.1 Ethical approval

This experiment complied with the relevant national animal welfare and ethics regulations, approved by the Animal Ethics Welfare Committee (AEWC) of Sichuan Agricultural University (ethical approval code: SICAU 2023314113).

### 2.2 Animals, diets and experimental design

**Figure 1:**
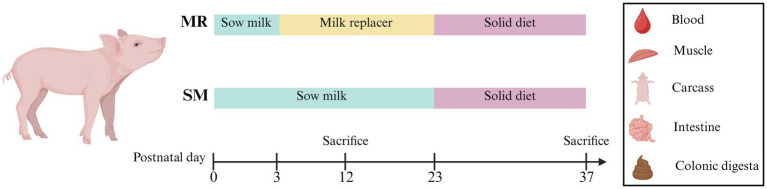
The overview of experimental design.

A total of forty 2-day-old piglets (Landrace × Yorkshire genotype, 4 piglets per litter, 2 males and 2 females, initial body weight 2.02 ± 0.06 kg) were selected from 10 healthy sows with similar parities (2, 3) and litter sizes (12, 13). The piglets were then randomly allocated to two groups (1): artificially reared with milk replacer (MR) or (2) sow-reared with sow milk (SM), with 10 replicates per group and two body weight (BW)-paired piglets per replicate. The experimental design is shown in Figure 1. All piglets were housed with their dams and allowed free access to sow milk for 48 h. After this period, the piglets were randomly assigned to either the MR or SM group. SM-fed piglets were maintained with the sow until postnatal day 23 (PND 23), during which the sows continued to nurse the entire litter throughout the lactation period. In contrast, MR-fed piglets were separated from the sows after 48 h of suckling and transported to the nursery room, where they were housed in metabolic crates (0.6 × 0.9 × 0.6 m) in pairs until PND 23. Their daily milk replacer intake was recorded. To minimize the influence of environmental microbes, feces from the corresponding sows were collected from the sow pen every 2 days and smeared onto the floor of the crates where the MR piglets were raised. Throughout the experimental period, piglets were monitored for diarrhea three times daily at 08:00, 14:00, and 20:00. Fecal consistency was scored by two trained researchers using a 4-point scale as follows: 0 = Normal, firm feces; 1 = Soft, partially formed feces; 2 = Loose, semi-liquid feces; 3 = Watery, liquid feces. The diarrhea index was calculated using the following formula: Diarrhea Index = *Σ* (Diarrhea Scores) / (Number of Piglets × Days of Observation × Daily Scoring Frequency). This method of diarrhea assessment was adapted from previous studies (23). Piglets’ health was monitored daily, with veterinary care provided as needed. Housing conditions adhered to animal welfare guidelines, with a temperature range of 28–31°C, humidity between 60 and 70%, and a 12 h light/dark cycle, to minimize environmental stress (24). After 10 days of feeding with MR or SM, 10 piglets from each group were slaughtered and sampled at PND 12. The remaining MR- and SM-fed piglets were weaned at PND 23 and fed the same solid diet until PND 37. SM-fed piglets were transported to the room where the MR-fed piglets were housed after weaning and were fed the same solid diet. After weaning, all piglets were housed individually and scored for diarrhea three times a day. Housing conditions were maintained with a room temperature of 26–28°C, humidity ranging from 60 to 70%, and a 12 h light/dark cycle. At the end of the experiment, piglets were slaughtered and sampled at PND 37 (*n* = 10). SM-fed piglets suckled their dams until PND 23, with litter sizes adjusted to 7–8 piglets per sow to ensure adequate milk intake for the study piglets. MR-fed piglets were allowed free access to milk replacer and water from PND 3 to PND 23. Each crate was equipped with feeding equipment, and the piglets received milk from the metabolic crate nipple. MR powder was dissolved in approximately 40°C warm water (dry matter content: 20%) and added to the fixed beaker of the metabolic crate seven times a day. To ensure balanced intake between individuals, daily milk replacer consumption was monitored for each piglet. If significant discrepancies in intake were observed, adjustments were made to encourage more uniform consumption across all MR-fed piglets. The composition and nutrient levels of the milk replacer are shown in Table 1. After weaning at PND 23, all piglets were fed the same solid diet ad libitum until PND 37. The composition and nutrient levels of the solid diet are shown in Table 2.

**Table 1:** Composition and nutrient levels of milk replacer for neonatal piglets.

Ingredients	Percentage, %
Lactose	15.54
Skimmed milk powder	19.88
Whey protein concentrate (73% CP)	21.60
Casein	6.60
Coconut oil	25.00
Sucrose	3.00
Glucose	2.00
Calcium hydrogen phosphate	1.18
Calcium citrate	3.20
Citrate	1.00
Premix^1^	1.00
Total	100.00
Calculated composition, %	
Digestible energy, Mcal/kg	4.759
Crude protein	28.00
Ether extract	25.42
Lactose	25.34
Calcium	1.00
Total phosphorus	0.71
Total lysine	2.66
Total methionine	0.69
Total methionine + cystine	1.21
Total threonine	1.55
Total tryptophan	0.50

^1Provided per kg of diet: VA 15,000 IU; VD3 5,000 IU; VE 40 IU; VK3 5.00 mg; VB1 5.00 mg; VB2 12.50 mg; VB6 6.00 mg; VB12 0.06 mg; D-biotin 0.25 mg; D-pantothenic acid 25.00 mg; folic acid 2.50 mg; nicotinamide 50.00 mg; Zn 100.00 mg; Cu 6.00 mg; Fe 100.00 mg; Mn 4.00 mg; I 0.14 mg; Se 0.30 mg.^

**Table 2:** Composition and nutrient levels of nursery diet.

Ingredients	Percentage, %
Extruded corn	40.70
Extruded soybeans	7.60
Enzymatic hydrolysis of soybeans	10.00
Soy protein concentrate	2.00
Whey powder	15.00
Whole milk powder	10.00
Fishmeal	4.00
Coconut oil	3.00
Glucose	4.00
L–lysine hydrochloride	0.49
DL–Methionine	0.20
L–threonine	0.18
L–tryptophan	0.16
Choline chloride	0.16
Limestone	0.88
Calcium hydrogen phosphate	0.24
Sodium chloride	0.40
Acidifier	0.50
Premix^1^	0.49
Total	100.00
Calculated composition, %	
Digestible energy, Mcal/kg	3.78
Crude protein	19.01
Ether extract	9.29
Calcium	0.83
Available phosphorus	0.43
SID Lysine	1.43
SID methionine	0.52
SID methionine + cystine	0.78
SID threonine	0.84

^1Provided per kg of diet: VA 15,000 IU; VD3 5,000 IU; VE 40 IU; VK3 5.00 mg; VB1 5.00 mg; VB2 12.50 mg; VB6 6.00 mg; VB12 0.06 mg; D-biotin 0.25 mg; D-pantothenic acid 25.00 mg; folic acid 2.50 mg; nicotinamide 50.00 mg; Zn 100.00 mg; Cu 6.00 mg; Fe 100.00 mg; Mn 4.00 mg; I 0.14 mg; Se 0.30 mg.^

### 2.3 Sample collection and measurements

Ten piglets per group were euthanized and samples were collected after overnight fasting at PND 12 and PND 37, respectively. Before slaughtering, piglets were weighed and blood samples were obtained from anterior vena cava. The head length, head-rump length, and abdominal circumference of the piglets were measured with a soft tape. Immediately after exsanguination, the entire left longissimus dorsi, semitendinosus, and psoas muscles were excised and weighed. In addition, samples of the longissimus dorsi, semitendinosus, psoas muscles, jejunum, ileum tissues, and colonic digesta were collected, quickly frozen in liquid nitrogen, and stored at −80°C. The carcasses of piglets, after removal of the head, trotter, tail and offal (while retaining the kidneys), were frozen and stored at −20°C.

### 2.4 Body chemical composition analysis

The frozen carcasses were cut into pieces and ground four times. The first three times were done through a 6-mm die in a commercial grinder (PE-180S Iron crushing machine, Xulang machine Company, Guangzhou, China), followed by a final grinding in a commercial grinder (Fangtai electrical appliances, Dongguan, China). The final ground samples were thoroughly mixed. Approximately 200 g of the final mixture, collected using the quartering method, was placed in an aluminum container and weighed. The samples were then freeze-dried to a constant weight in a freeze dryer (LAB-BL2, BiLon, Shanghai, China). After freeze-drying, all samples were weighed, ground, and mixed again in a commercial grinder. The water content was calculated by measuring the weight loss after freeze-drying. Dry matter content was determined according to the methods of AOAC (25). Crude protein content was determined by using a OLABO fully automatic Kjeldahl nitrogen analyzer (OLB9870A, OLABO, Jinan, China). Crude fat content was determined by the soxhlet extraction method (26). Ash content was determined based on GB_T23742-2009. Gross energy was determined by Parr6400 oxygen bomb calorimeter (Parr 6,400, Parr, Illinois, United States).

### 2.5 Plasma biochemistry analysis

Plasma samples stored at −20°C were centrifuged at 2500 rpm for 20 min, and the supernatant was used to measure metabolism-related indicators, including urea, glucose (GLU), total cholesterol (TC), low density lipoprotein cholesterol (LDL-C), high density lipoprotein cholesterol (HDL-C), non-esterified fatty acid (NEFA), triglyceride (TG) and complement 3 (C3) concentration by a fully automatic biochemical analyzer (Hitachi 3,100, Tokyo, Japan). For urea, GLU, TC, LDL-C, HDL-C, and TG, a defined volume of plasma was added to the respective reagent. After mixing, the reaction mixtures were incubated at 37°C for a specified duration, typically ranging from 10 to 20 min. After incubation, absorbance values were measured at appropriate wavelengths (e.g., 340 nm for urea, 500 nm for GLU, and 520 nm for HDL-C) using the analyzer. Concentrations were calculated based on standard calibration curves provided with each assay kit (Urea CH0101051, GLU CH0101102, TC CH0101152, LDL-C CH0103162, HDL-C CH0101161, TG CH0101151, Maccura, Chengdu, China). For NEFA, a plasma sample was mixed with a specific reagent according to the protocol outlined in the Sjodax kit (RB30951, Sjodax, Beijing, China). The reaction was conducted at 37°C for 10 min, after which the absorbance was measured at 550 nm to determine the NEFA concentration. C3 concentration was measured using the Iprocom C3 kit (C30001S, Iprocom, Anhui, China). Plasma samples were mixed with the C3-specific reagent, incubated at 37°C, and the absorbance was measured at 450 nm to quantify the C3 levels.

### 2.6 Enzyme activity detection

Jejunal tissue samples were homogenized in pre-cooled 0.9% saline at 4°C for further analysis. The homogenates were processed for protein quantification using the Coomassie Brilliant Blue method. Protein concentration was determined using the Coomassie Brilliant Blue Protein Assay Kit (A045-2, Nanjing Jiancheng, Nanjing, China), according to the manufacturer’s instructions. An aliquot of the homogenized jejunal tissue (typically 10 ~ 20 μL) was mixed with 200 μL of Coomassie Brilliant Blue reagent provided in the kit. The mixture was incubated at room temperature for 5 ~ 10 min, allowing the Coomassie dye to bind to the protein. After incubation, the absorbance of each sample was measured at 595 nm using a spectrophotometer. The absorbance values were used to determine the protein concentration by comparing them to a standard curve prepared with known concentrations of bovine serum albumin (BSA) provided with the kit.

The activities of lactase, maltase, and sucrase in jejunal tissue were measured according to the instructions provided by the Nanjing Jiancheng kit (lactase A082-1-1, maltase A082-3-1, sucrase A082-2-1, Nanjing Jiancheng, Nanjing, China). The reaction buffer and substrates provided in the assay kits were prepared according to the manufacturer’s guidelines. All reagents were mixed thoroughly and equilibrated to room temperature before use. The tissue homogenates were diluted appropriately to ensure enzyme activity fell within the linear range of the assay. A suitable volume of the homogenate (typically 10 ~ 20 μL) was transferred into the reaction wells. For each enzyme activity assay (lactase, maltase, or sucrase), a defined volume of enzyme substrate was added to the sample. The reaction mixtures were incubated at 37°C for the time specified in the kit protocol (typically 10 ~ 20 min). The reaction was stopped by adding a stop solution instructed by the kit. The enzymatic activity was measured by determining the absorbance at the specific wavelength provided in the kit’s instructions (e.g., 420 nm for lactase, 405 nm for maltase, and 510 nm for sucrase). Enzyme activity was quantified based on the absorbance values, using the provided equation in the kit.

The activities of succinate dehydrogenase (SDH), malate dehydrogenase (MDH), and lactate dehydrogenase (LDH) in the tissue samples were measured using commercially available assay kits (SDH A022-1-1, MDH A021-2-1, and LDH A020-1, Nanjing Jiancheng, Nanjing, China), according to the manufacturer’s instructions. The reagents and buffers provided in the kits were prepared according to the manufacturer’s guidelines. All solutions were mixed thoroughly and equilibrated to room temperature before use. A suitable volume of the tissue homogenate (usually 10 ~ 20 μL) was added to the reaction mixture, ensuring that the enzyme concentrations were within the optimal range for measurement. The reaction mixtures were incubated at 37°C for the time specified in the respective kit protocols. This step allowed the enzymatic reactions to proceed, with the substrates being converted to their corresponding products. After incubation, the reactions were stopped by the addition of a stop solution, as specified by the kit instructions, to halt further enzyme activity. The enzyme activity was determined by measuring the absorbance at the specific wavelength recommended for each assay (e.g., 600 nm for SDH, 340 nm for MDH, and 440 nm for LDH). Enzyme activities were calculated from the absorbance values using standard curves provided with the kits. The results were expressed as units per milligram of protein (U/mg protein).

### 2.7 Immunofluorescence analysis

Immunofluorescence double-labeling technology was performed on longissimus dorsi muscle fixed with 4% paraformaldehyde by Servicebio (Servicebio, Wuhan, China). The specific operation involved dewaxing of the prepared longissimus dorsi muscle paraffin sections, followed by antigen retrieval. Sections were blocked with 3% bovine serum albumin (GC305010, Servicebio, Wuhan, China) for 30 min at room temperature. Slow MyHC primary antibody (GB112131, Servicebio, Wuhan, China) was added and incubated overnight at 4°C. Slow MyHC secondary antibody (Alexa Fluor 488 labeled goat anti-rabbit IgG, GB25303, Servicebio, Wuhan, China) was then added and incubated at room temperature for 50 min. The corresponding iF488-Tyramide dye (G1231, Servicebio, Wuhan, China) was added and incubated at room temperature for 10 min in the dark. Following the same experimental steps as above, the primary antibody for Fast MyHC (GB112130, Servicebio, Wuhan, China) and the secondary antibody (HRP-labeled goat anti-rabbit IgG, GB23303, Servicebio, Wuhan, China) were added and incubated. Then, add the corresponding iF555-Tyramide dye (G1233-25UL, Servicebio, Wuhan, China) and incubate at room temperature for 10 min in the dark. Nuclei were counterstained with DAPI (G1012, Servicebio, Wuhan, China). Finally, the slides were mounted with anti-fluorescence quenching mounting medium (G1401, Servicebio, Wuhan, China). Fluorescence imaging of the cells was performed using a Nikon upright fluorescence microscope (NIKON ECLIPSE C1, Nikon, Tokyo, Japan). Through ultraviolet irradiation, the cell nucleus appeared blue, the fast muscle fiber cells appeared red, and the slow muscle fiber cells appeared green.

### 2.8 Periodic acid-shiff staining

First, ileum tissue samples fixed in 4% paraformaldehyde were rinsed with running water for 30 min. Then, the tissue blocks were trimmed and subjected to dehydration in a series of ethanol solutions: 75% ethanol for 6 h, 85% ethanol for 10 h, 95% ethanol for 4 h, absolute ethanol I for 2 h, and anhydrous Ethanol II for 2 h. The samples were then cleared in xylene I for 20 min, followed by xylene II for 15 min, and immersed in wax for 3 h before being embedded in paraffin. Next, the tissue blocks were sectioned into 5 μm thick slices with a microtome (Leica RM2235, Leica, Wetzlar, Germany). The slices were flattened in warm water, placed onto glass slides, and then baked at 60°C for at least 2 h. After dewaxing with xylene, the sections were rinsed with running water for 20 min. They were then oxidized with 1% periodic acid for 5 min, rinsed with running water for 5 min, and stained with PAS solution for 10–30 min at 37°C. After rinsing with running water for 5 min, the nuclei were stained with hematoxylin for 10–30 s, differentiated with hydrochloric acid and alcohol, dehydrated through a gradient alcohol series, made transparent with xylene, and finally sealed with resin. The height of 10 complete villi and the depth of 10 crypts were measured. The villus-to-crypt ratio was calculated, and the number of goblet cells was counted using Image-Pro Plus 6.0 software (Media Cybernetics, Maryland, United States).

### 2.9 Total RNA extraction and real-time PCR

First, ileum and muscle tissue RNA was extracted using RNA isolater Total RNA Extraction Reagent (Vazyme, Nanjing, China) according to the instructions. Then the reverse transcription of RNA was performed using a kit with 4 × gDNA wiper Mix and 5 × HiScript III RT SuperMix (Vazyme, Nanjing, China) according to the instructions. The relative mRNA expression of genes (Nuclear Factor Kappa B (*NF-κB*), Interleukin-1 beta (*IL-1β*), Interleukin-6 (*IL-6*), Tumor Necrosis Factor-alpha (*TNF-α*), Interleukin-10 (*IL-10*), Zonula Occludens-1 (*ZO-1*), *Occludin*, *Claudin*, Toll-Like Receptor 9 (*TLR-9*), Toll-Like Receptor 4 (*TLR-4*), Myeloid Differentiation Primary Response 88 (*MyD88*), TNF Receptor Associated Factor 6 (*TRAF6*), Toll-Interacting Protein (*TOLLIP*), Myosin Heavy Chain I (*MyHC-I*), Myosin Heavy Chain IIb (*MyHC-IIb*), Myosin Heavy Chain IIa (*MyHC-IIa*), Myosin Heavy Chain IIx (*MyHC-IIx*)) was determined using real-time fluorescence quantification (Real-time PCR) method (27). The primers used in this experiment are listed in Table 3.

**Table 3:** Sequences of primer for the target genes.

Genes	Primer sequence (5′–3′)	Product (bp)	GenBank accession
*IL*-*6*	F: TGCAGTCACAGAACGAGTGG	116	NM_214399.1
	R: CAGGTGCCCCAGCTACATTAT		
*IL*-*10*	F: AATCTGCTCCAAGGTTCCCG	224	NM_214041.1
	R: TGAACACCATAGGGCACACC		
*IL*-*1β*	F: TCTGCCCTGTACCCCAACTG	64	NM_214055.1
	R: CCAGGAAGACGGGCTTTTG		
*TNF*-*α*	F: CCACGTTGTAGCCAATGTCA	395	NM_214022.1
	R: CAGCAAAGTCCAGATAGTCG		
*NF*-*κB*	F: AGTACCCTGAGGCTATAACTCG	146	NM_001114281.1
	R: TGAGAAGTCCATGTCCGCAAT		
*Claudin*-*1*	F: ACTGGCTGGGCTGCTGCTTCTCT	101	NM_001244539.1
	R: GGATAGGGCCTTGGTGTTGGGTAA		
*Occludin*	F: CAGCAGCAGTGGTAACTTGG	110	NM_001163647.2
	R: CCGTCGTGTAGTCTGTCTCG		
*ZO*-*1*	F: CCGCCTCCTGAGTTTGATAG	189	XM_013993251.1
	R: CAGCTTTAGGCACTGTGCTG		
*TLR*-*9*	F: AATCCAGTCGGAGATGTTTGCT	79	NM_213958.1
	R: GACCGCCTGGGAGATGCT		
*MyD88*	F: GTGCCGTCGGATGGTAGTG	65	NM_001099923.1
	R: TCTGGAAGTCACATTCCTTGCTT		
*TLR*-*4*	F: CAGATAAGCGAGGCCGTCATT	113	NM_001113039.2
	R: TTGCAGCCCACAAAAAGCA		
*TRAF6*	F: CCAGAGACCCACAATCCCAC	125	NM_001105286.1
	R: TGGAGACCTCACAGCGTACT		
*TOLLIP*	F: CCCGCGCTGGAATAAGG	74	NM_001315800.1
	R: CATCAAAGATCTCCAGGTAGAAGGA		
*MyHC-I*	F: AAGGGCTTGAACGAGGAGTAGA	115	NM_213855.2
	R: TTATTCTGCTTCCTCCAAAGGG		
*MyHC-IIb*	F: CAAAATCCTTCGCATCCAACTT	67	NM_001123141.1
	R: CTCAGCAATTTTACGGTCAATCTC		
*MyHC-IIa*	F: GCTGAGCGAGCTGAAATCC	137	NM_214136.1
	R: ACTGAGACACCAGAGCTTCT		
*MyHC-IIx*	F: AGAAGATCAACTGAGTGAACT	149	NM_001104951.2
	R: AGAGCTGAGAAACTAACGTG		
*β*-*actin*	F: GGCGCCCAGCACGAT	66	XM_021086047.1
	R: CCGATCCACACGGAGTACTTG		

IL-6: Interleukin 6, IL-10: Interleukin 10, IL-1β: Interleukin–1β, TNF-α: Tumor necrosis factor-α, NF-κB: Nuclear factor kappa-B, ZO-1: tight junction protein 1, TLR-9: Toll-like receptor 9, MyD88: Myeloid differentiation factor 88, TLR-4: Toll-like receptor 4, TRAF6: TNF receptor associated factor 6, TOLLIP: Toll interacting protein, MyHC-I: Myosin Heavy Chain I, MyHC-IIb: Myosin Heavy Chain IIb, MyHC-IIa: Myosin Heavy Chain IIa, MyHC-IIx: Myosin Heavy Chain IIx.

### 2.10 DNA extraction and 16S rRNA gene sequencing

DNA from colonic digesta was extracted using the Tiangen magnetic bead extraction kit (DP712, TianGen, Beijing, China) according to the manufacturer’s instructions. The extracted DNA was examined on a 1% agarose gel, and its concentration and purity were determined using a NanoDrop 2000 spectrophotometer (Thermo Scientific, Waltham, United States). The DNA was then diluted to 1 ng/μL with sterile water and used as PCR template. Primers (F: 5’-GTGCCAGCMGCCGCGGTAA-3′; R: 5’-GGACTACHVGGGTWTCTAAT-3′) were used to amplify the V3–V4 hypervariable region of the 16S rRNA gene. PCR was performed using New England Biolabs’s Phusion® High-Fidelity PCR Master Mix (M0532S, New England Biolabs, Massachusetts, United States) and Phusion® High-Fidelity DNA polymerase (M0530S, New England Biolabs, Massachusetts, USA) to ensure amplification efficiency and accuracy. The total PCR reaction volume was 30 μL, containing 15 μL Phusion Master Mix (New England Biolabs, Massachusetts, USA), 0.2 μL each of Primer F (1 μM) and Primer R (1 μM), and 10 μL gDNA (1 ng/μL). The mixture was adjusted to 30 μL with ddH_2_O. The specific PCR reaction program was as follows: initial denaturation at 98°C for 1 min, followed by 30 cycles of 98°C for 10 s, 50°C for 30 s, and 72°C for 30 s, with a final extension at 72°C for 5 min. The PCR products were purified using 2% agarose gel electrophoresis, and the target bands were recovered with the MinElute PCR Purification Kit (Qiagen, Dusseldorf, Germany). The library was constructed using the NEBNext® UltraTM II DNA Library Prep Kit (E7645, New England Biolabs, Massachusetts, United States), and quantified using Qubit and Q-PCR. After the library was quantified, sequencing was performed using the NovaSeq 6,000 platform (Illumina Novaseq6000, Illumina, San Diego, CA, United States). For bioinformatics analysis, the final ASVs (amplicon sequence variants) were obtained by denoising valid data with DADA2 (28) and filtering out sequences with an abundance less than 5. Species richness and evenness within samples were assessed based on abundance, and Alpha diversity and Venn plot analysis of ASVs were performed. In addition, the PCoA (Principal Coordinates Analysis) and LEfSe (Linear Discriminant Analysis Effect Size) were used to explore the differences in microbial community structure between groups.

### 2.11 Short chain fatty acids determination

Approximately 0.5 g of colonic digesta was accurately weighed. The content of short-chain fatty acids was determined by a gas chromatograph (GC CP3800, Varian Medical Systems, Palo Alto, California, United States). Specific experimental operations were carried out according to our previous study (29).

### 2.12 Statistical analysis

The variance homogeneity and normality of the data were analyzed using the UNIVARIATE and Shapiro–Wilk methods. For data that met the assumptions of normality and homogeneity of variance, differences between groups were analyzed using the t-test. For data that did not meet these assumptions, the non-parametric Mann–Whitney U test was employed. All statistical analyses were performed using SAS 9.4 (SAS Institute, Inc., Cary, NC, United States). All data are expressed as mean ± SEM. Results are considered statistically significant when *p* < 0.05, and considered as tendencies when 0.05 ≤ *p* < 0.10. The top 6 and 20 species with the highest abundance at the phylum and genus level, respectively, were selected to generate a cumulative histogram of species relative abundance. We performed PCoA analysis based on Unweighted Unifrac distance, and selected the combination of principal coordinates with the largest contribution for drawing. The differences in the microbial community structure between groups were identified using LEfSe analysis with an LDA Score of 4. The Spearman correlation analysis was used to determine the relationship between the relative abundance of the genus and the concentration of short chain fatty acids concentration. Results with *p* < 0.05 were considered statistically significant. Histograms and heatmaps were generated with GraphPad Prism 9.5.0 (San Diego, CA, United States).

## 3 Results

### 3.1 Growth performance and diarrhea index

Compared to SM-fed piglets, MR-fed piglets exhibited lower ADG (*p* = 0.08) and a higher diarrhea index (*p* < 0.01) from PND 3 to 12 (Table 4). Conversely, MR-fed piglets showed higher ADFI and ADG from PND 24 to 37 (*p* < 0.01), along with a lower ratio of ADFI to ADG (F:G) (*p* < 0.01) and a lower diarrhea index (*p* = 0.08) compared to SM-fed piglets.

**Table 4:** Effects of MR and SM on growth performance and diarrhea index of piglets.

	Diet		*p*-value
	MR	SM	
PND 3–12			
Initial BW, kg	2.03 ± 0.08	2.00 ± 0.06	0.76
Final BW, kg	3.87 ± 0.12	4.04 ± 0.06	0.34
ADG, g/d	184 ± 6	204 ± 6	0.08
Diarrhea index	0.83 ± 0.07	0.39 ± 0.03	<0.01
PND 12–23			
Initial BW, kg	3.77 ± 0.16	3.79 ± 0.14	0.92
Final BW, kg	5.82 ± 0.18	5.80 ± 0.18	0.95
ADG, g/d	186 ± 4	183 ± 7	0.66
Diarrhea index	0.50 ± 0.06	0.47 ± 0.06	0.69
PND 23–37			
Initial BW, kg	5.82 ± 0.18	5.80 ± 0.18	0.95
Final BW, kg	9.09 ± 0.31	7.02 ± 0.43	<0.01
ADG, g/d	233 ± 11	87 ± 22	<0.01
ADFI, g/d	338 ± 12	174 ± 30	<0.01
F:G	1.46 ± 0.03	2.18 ± 0.15	<0.01
Diarrhea index	0.45 ± 0.08	0.93 ± 0.23	0.08

MR, milk replacer, SM, sow milk, PND, postnatal day; ADG, average daily gain; ADFI, average daily feed intake; F:G, ratio of ADFI to ADG. Data are presented as the mean ± SEM (*n* = 10).

### 3.2 Body composition and body mass index

**Figure 2:**
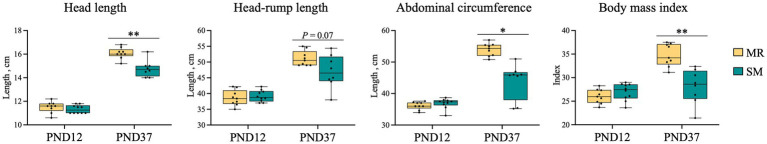
Effects of MR and SM on the body mass index of piglets. PND: postnatal day, Body mass index = body weight/(head-rump length)^2^. Data are presented as the mean ± SEM. ^*^p < 0.05, ^**^p < 0.01.

Compared to SM-fed piglets, MR-fed piglets had higher head length, abdominal circumference, and body mass index (*p* < 0.05), as well as head-rump length (*p* = 0.07) on PND 37 (Figure 2). On PND 12, MR-fed piglets had lower carcass weight (*p* < 0.01) and higher crude protein content (*p* = 0.05). On PND 37, however, MR-fed piglets showed higher carcass weight (*p* < 0.01, Table 5). Additionally, MR-fed piglets had higher water content, but lower body fat, dry matter, and GE on both PND 12 and PND 37 (*p* < 0.01).

**Table 5:** Effects of MR and SM on body composition of piglets.

	PND 12			PND 37		
	MR	SM	*P*-value	MR	SM	*P*-value
Carcass weight, kg	2.23 ± 0.08	2.70 ± 0.07	<0.01	5.37 ± 0.23	3.85 ± 0.35	<0.01
Water, %	75.65 ± 0.46	66.42 ± 0.80	<0.01	70.09 ± 0.39	65.58 ± 0.57	<0.01
Dry matter, %	24.35 ± 0.46	33.58 ± 0.80	<0.01	29.91 ± 0.39	34.42 ± 0.57	<0.01
Crude protein, %	15.06 ± 0.23	14.51 ± 0.14	0.05	15.83 ± 0.14	16.12 ± 0.46	0.56
Fat, %	5.39 ± 0.35	14.31 ± 0.63	<0.01	10.13 ± 0.33	13.53 ± 0.42	<0.01
Ash, %	2.75 ± 0.07	2.71 ± 0.05	1.00	2.73 ± 0.05	2.90 ± 0.14	0.63
GE, MJ/kg	5.58 ± 0.21	9.35 ± 0.30	<0.01	7.72 ± 0.13	9.31 ± 0.24	<0.01

MR, milk replacer; SM, sow milk; PND, postnatal day; GE, gross energy. Data are presented as mean ± SEM (*n* = 10).

### 3.3 Muscle fiber type

**Figure 3:**
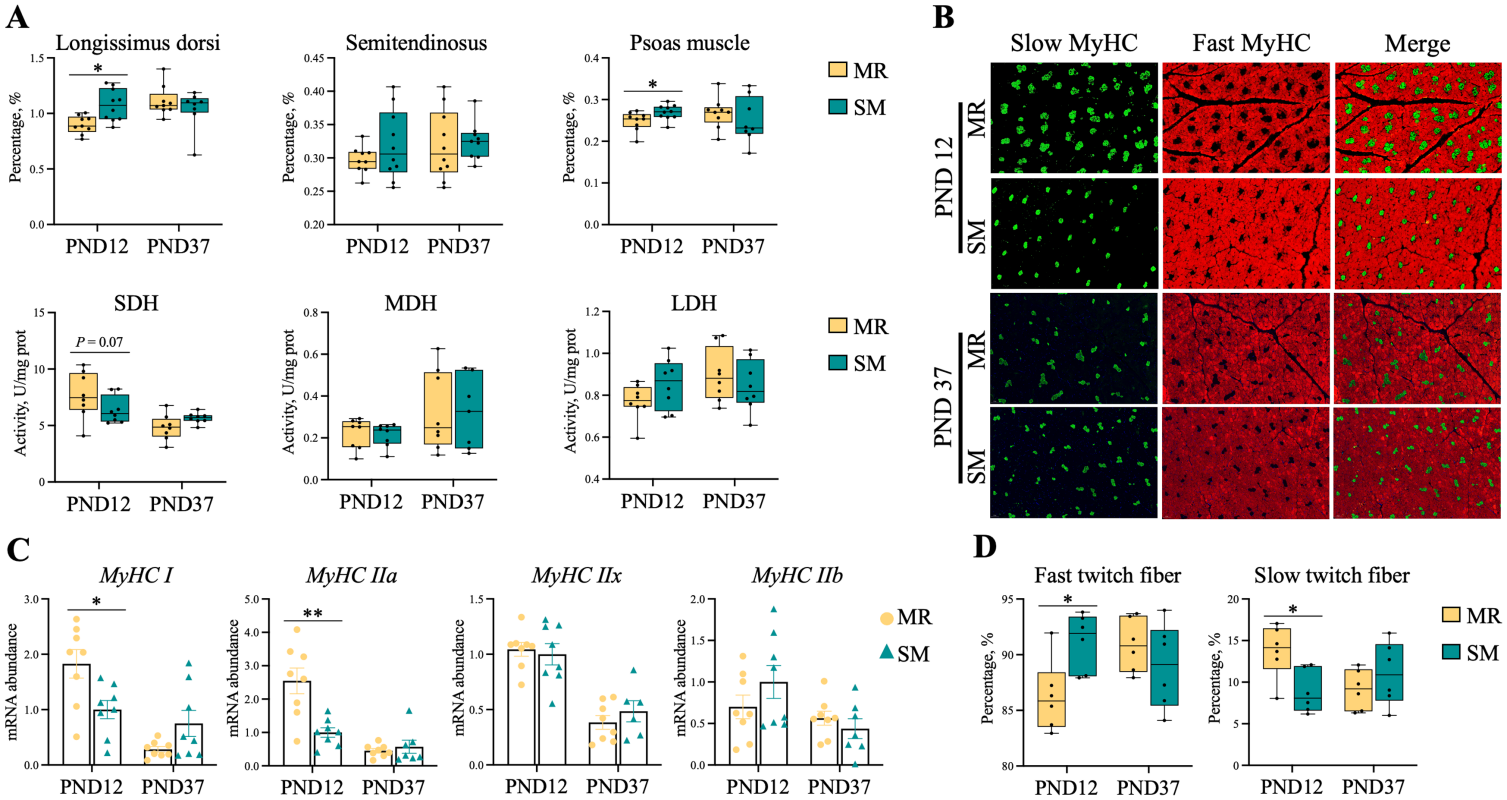
Effects of MR and SM on muscle development in piglets. PND: postnatal day. **(A)** The skeletal muscle percentage of body weight and activity of muscle energy metabolism enzymes. Muscle percentage (%) = (muscle weight/body weight) * 100. SDH, succinate dehydrogenase; MDH, malate dehydrogenase; LDH, lactate dehydrogenase. **(B)** Immunofluorescence analysis (green represents slow-twitch fibers and red represents fast-twitch fibers. Bar = 50 μm). **(C)** Relative mRNA expressions of *MyHC-I*, *MyHC-IIa*, *MyHC-IIx* and *MyHC-IIb*. **(D)** The percentage of fast-twitch and slow-twitch. Data are presented as the mean ± SEM. ^*^p < 0.05, ^**^p < 0.01.

Compared to SM-fed piglets, MR-fed piglets had a lower percentage of longissimus dorsi and psoas muscles relative to body weight on PND 12 (*p* ≤ 0.04; Figure 3A). The visual immunofluorescence pictures of longissimus dorsi are shown in Figure 3B, where MR-fed piglets had a lower percentage of fast twitch fibers, but a higher percentage of lower twitch fibers on PND 12 (*p* < 0.05; Figure 3D). Additionally, MR-fed piglets had higher relative mRNA expressions of *MyHC-I* and *MyHC-IIa* on PND 12 (*p* < 0.05; Figure 3C), but lower SDH activity on PND 37 (*p* = 0.07; Figure 3A).

### 3.4 Plasma biochemical indicators

**Figure 4:**
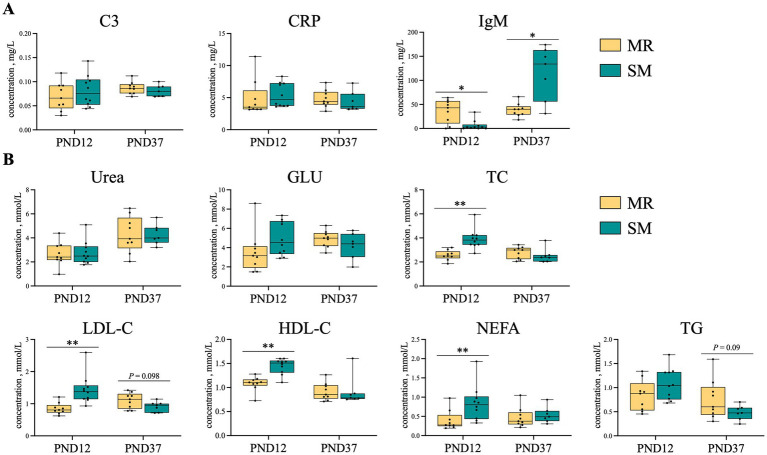
Shows the effects of MR and SM on plasma biochemical indicators in piglets. **(A)** Immunological indicators. **(B)** Metabolism-related indicators. C3: complement 3, CRP: C reactive protein, GLU: glucose, TC: total cholesterol, LDL-C: low density lipoprotein cholesterol, HDL-C: high density lipoprotein cholesterol, NEFA: non-esterified fatty acid, and TG: triglyceride. Data are presented as the mean ± SEM. ^*^p < 0.05, ^**^p < 0.01.

Compared to SM-fed piglets, MR-fed piglets had lower concentrations of TC, LDL-C, HDL-C, and NEFA on PND 12 (*p* < 0.01), but had higher IgM concentration (*p* < 0.05; Figures 4A,B). On PND 37, MR-fed piglets had lower IgM concentration (*p* < 0.05), but higher LDL-C (*p* = 0.09) and TG (*p* = 0.09) concentrations compared to SM-fed piglets (Figures 4A,B).

### 3.5 Intestinal morphology and enzyme activity

**Figure 5:**
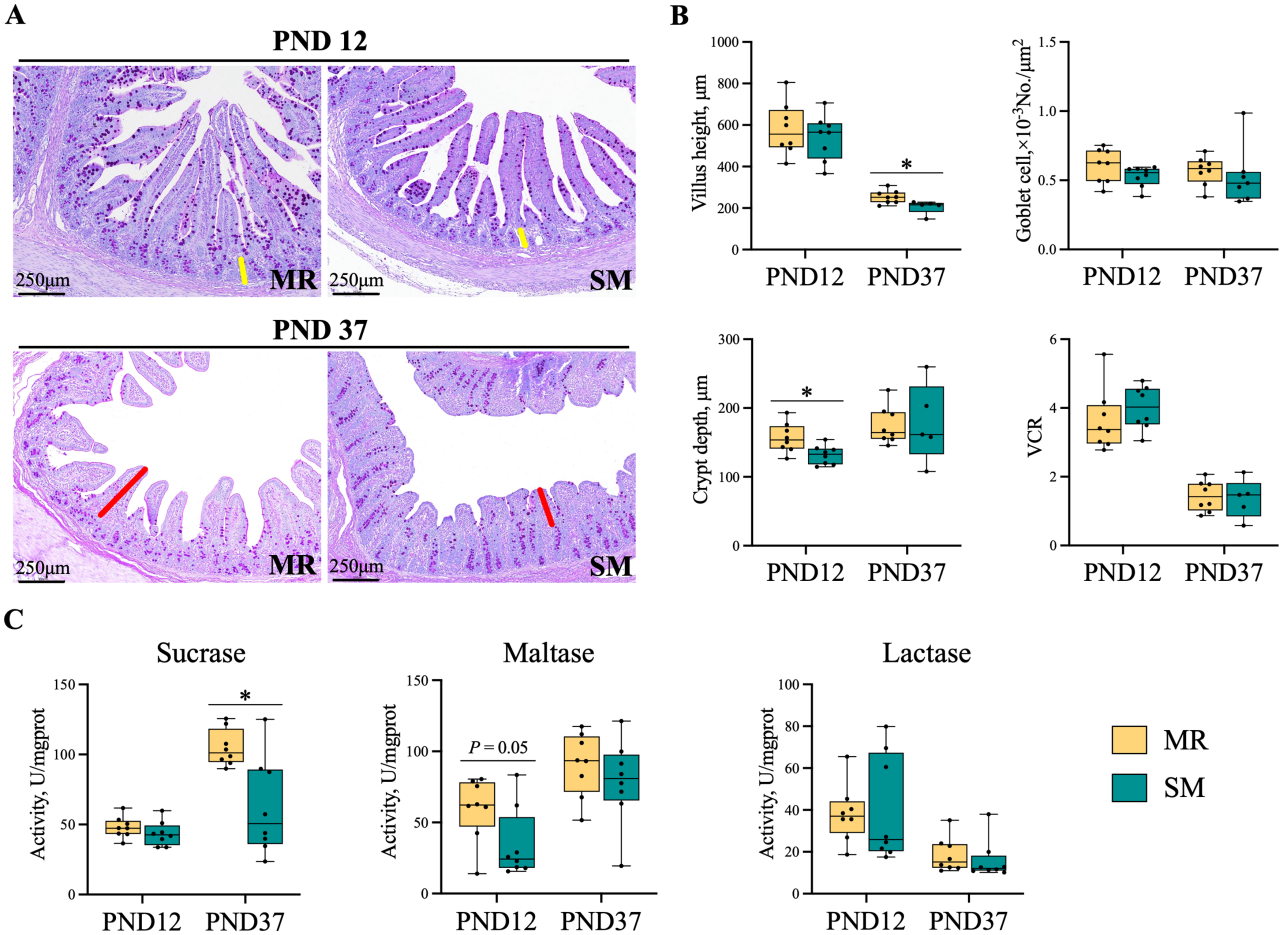
Effects of MR and SM on ileal morphology and jejunal disaccharidase activity in piglets. PND: postnatal day. **(A)** Periodic Acid-Shiff (PAS) stain: Yellow represents crypt depth, and red represents villus height. Bar = 250 μm. **(B)** Morphological evaluation of ileal mucosal epithelium. **(C)** Jejunal tissue disaccharidase activity. Data are presented as the mean ± SEM. ^*^p < 0.05.

The visual PAS staining pictures of ileal morphology are shown in Figure 5A. Compared to SM-fed piglets, MR-fed piglets had deeper crypt depth (*p* < 0.05) and higher maltase activity (*p* = 0.05) on PND 12 (Figures 5B,C). On PND 37, however, MR-fed piglets had higher villus height and sucrase activity (*p* < 0.05, Figures 5B,C).

### 3.6 Ileal gene expressions

**Figure 6:**
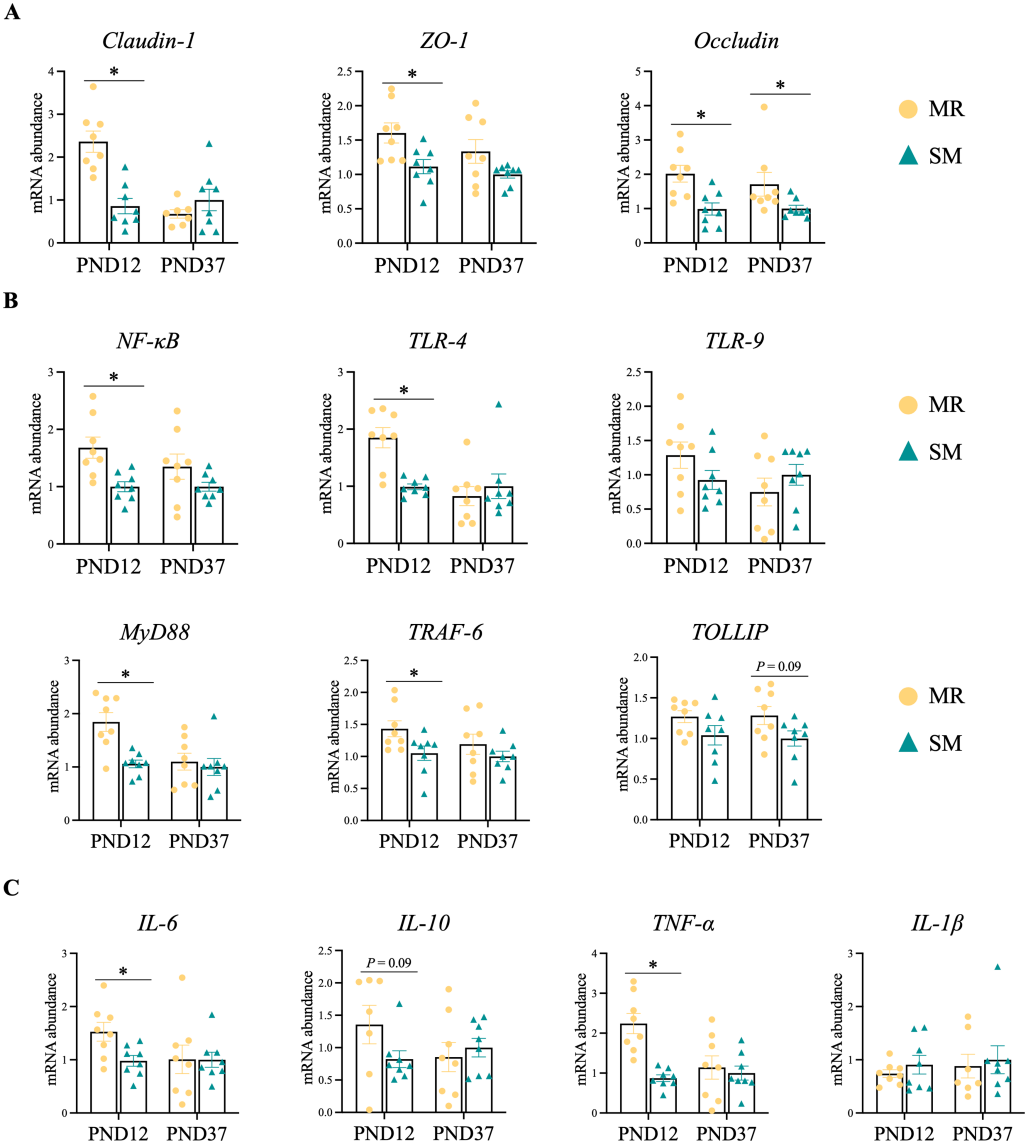
Effects of MR and SM on ileal gene expression in piglets. PND: postnatal day. **(A)** Relative mRNA expressions of barrier genes in ileal tissue. **(B)** Relative mRNA expressions of innate immunity genes in ileal tissue. **(C)** Relative mRNA expressions of inflammatory genes in ileal tissue. Data are presented as the mean ± SEM. ^*^p < 0.05.

Compared to SM-fed piglets, MR-fed piglets exhibited higher relative mRNA expressions of *Occludin*, *ZO-1*, *Claudin-1*, *IL-6*, *TNF-α*, *NF-κB*, *TLR-4*, *MyD88*, *TRAF-6* (*p* < 0.05), and *IL-10* (*p* = 0.09) on PND 12 (Figures 6A–C). On PND 37, however, MR-fed piglets showed higher relative mRNA expressions of *Occludin* (*p* < 0.05) and *TOLLIP* (*p* = 0.09; Figures 6A,B).

### 3.7 Microbiota composition

**Figure 7:**
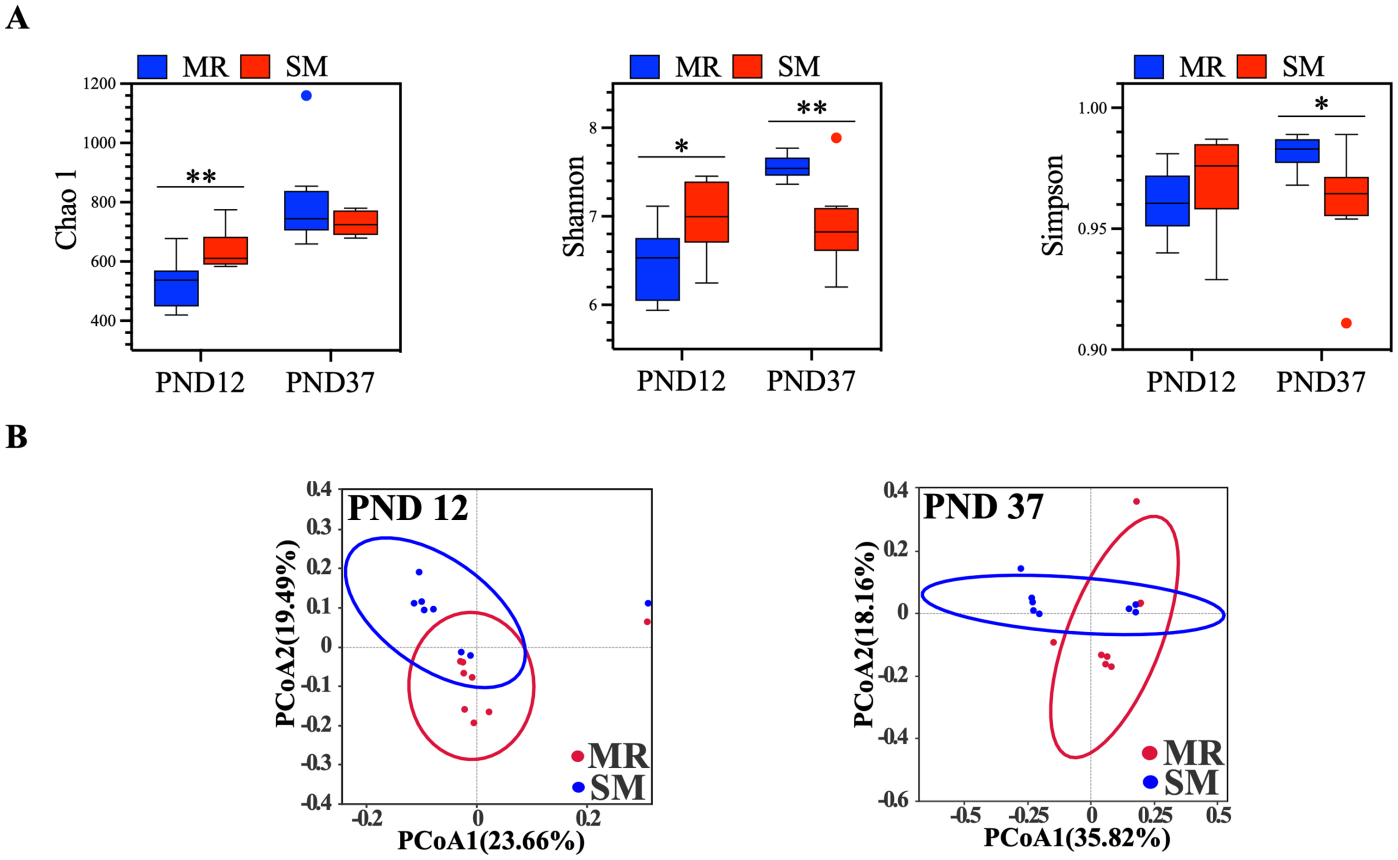
Effects of MR and SM on microbial alpha diversity index **(A)** and principal co-ordinates analysis **(B)** in piglets (*n* = 8). PND, postnatal day.

MR-fed piglets had significantly lower Chao1 richness estimator and Shannon diversity indices on PND 12, but higher Shannon and Simpson diversity indices on PND 37 compared to SM-fed piglets (Figure 7A). According to PCoA results, the variation explained by PC1 and PC2 was 23.66 and 19.49%, respectively, on PND 12, and 35.82 and 18.16%, respectively, on PND 37 (Figure 7B). Differences in the microbial community structure between the MR and SM groups were found based on the distance between samples.

**Figure 8:**
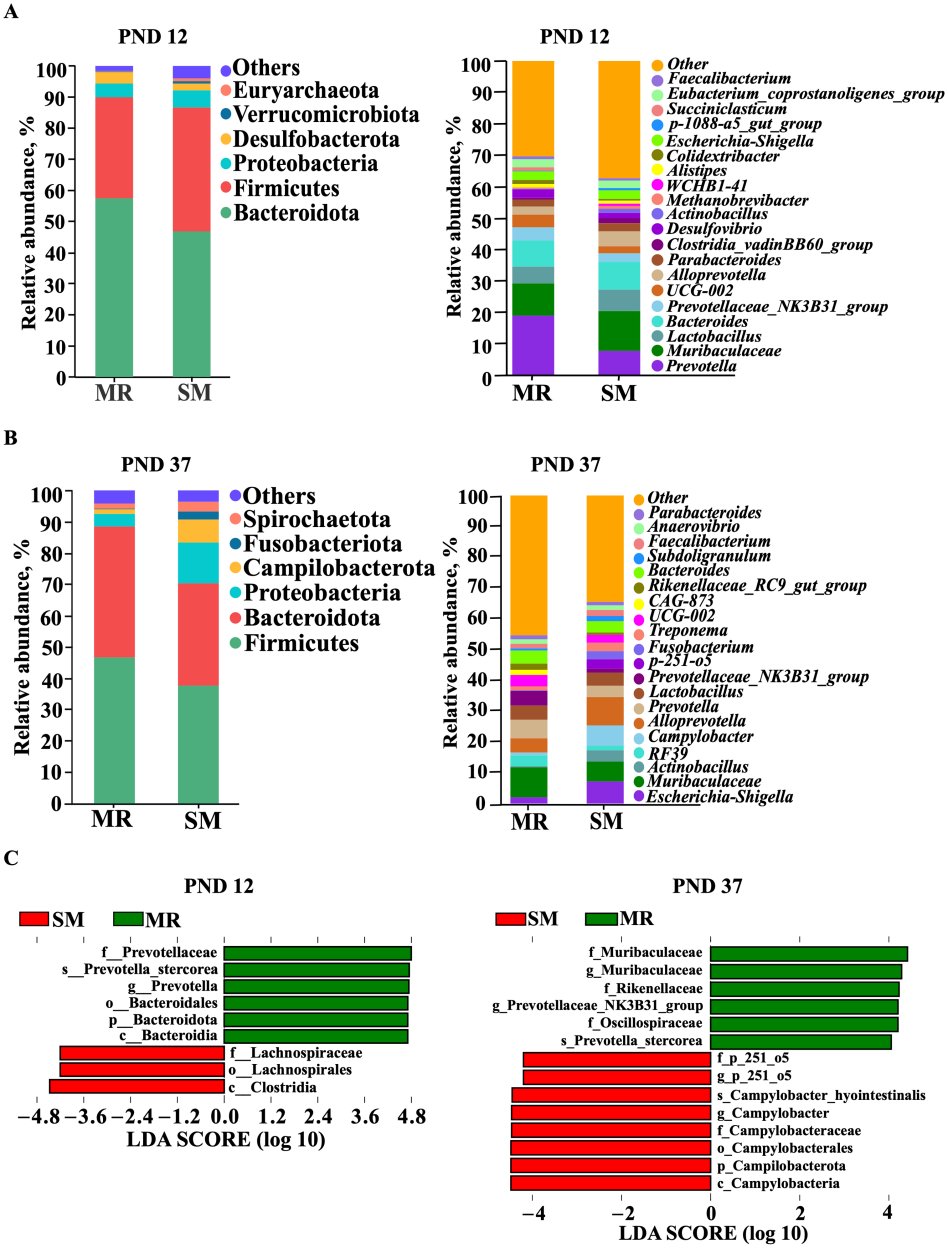
Effects of MR and SM on microbiota structure in piglets (*n* = 8). PND: postnatal day. **(A)** Most abundant species at phylum and genus level on PND 12. **(B)** Most abundant species at the phylum and genus levels on PND 37. **(C)** LDA value distribution histogram on PND 12 and PND 37. The histogram shows species with LDA scores>4, with the length of the bars representing the effect size of the different species (LDA score).

At the phylum level, the top four dominant bacterial groups on PND 12 were Bacteroidota (MR group: 57.68%, SM group: 46.95%), Firmicutes (MR group: 32.47%, SM group: 39.78%), Proteobacteria (MR group: 4.42%, SM group: 5.49%) and Desulfobacterota (MR group: 3.51%, SM group: 2.20%) (Figure 8A). On PND 37, the top four dominant bacterial groups were Firmicutes (MR group: 46.83%, SM group: 37.87%), Bacteroidota (MR group: 41.77%, SM group: 32.65%), Proteobacteria (MR group: 4.14%, SM group: 12.89%) and Campilobacterota (MR group: 1.45%, SM group: 7.42%) (Figure 8B).

At the genus level, the most dominant genera on PND 12 were *Prevotella*, *Muribaculaceae*, *Lactobacillus*, *Bacteroides*, *Prevotellaceae_NK3B31_group*, *UCG-002*, *Alloprevotella*, *Parabacteroides*, *Clostridia_vadinBB60_group* and *Desulfovibrio* (Figure 8A). On PND 37, the most dominant genera were *Escherichia-Shigella*, *Muribaculaceae*, *Actinobacillus*, *RF39*, *Campylobacter*, *Alloprevotella*, *Prevotella*, *Lactobacillus*, *Prevotellaceae_NK3B31_group* and *p-251-o5* (Figure 8B).

Analysis with the LEfSe algorithm revealed that *Prevotella* (Genus) was the dominant bacteria in the MR group, while Lachnospiraceae (Family) was the dominant bacteria in the SM group on PND 12 (Figure 8C). Additionally, Muribaculaceae (Family), *Prevotellaceae_NK3B31_group* (Genus), Oscillospiraceae (Family) and Rikenellaceae (Family) were identified as dominant bacteria in the MR group, while *p-251-o5* (Genus) and *Campylobacter* (Genus) were the dominant bacteria in the SM group on PND 37 (Figure 8C).

### 3.8 SCFAs concentration

**Figure 9:**
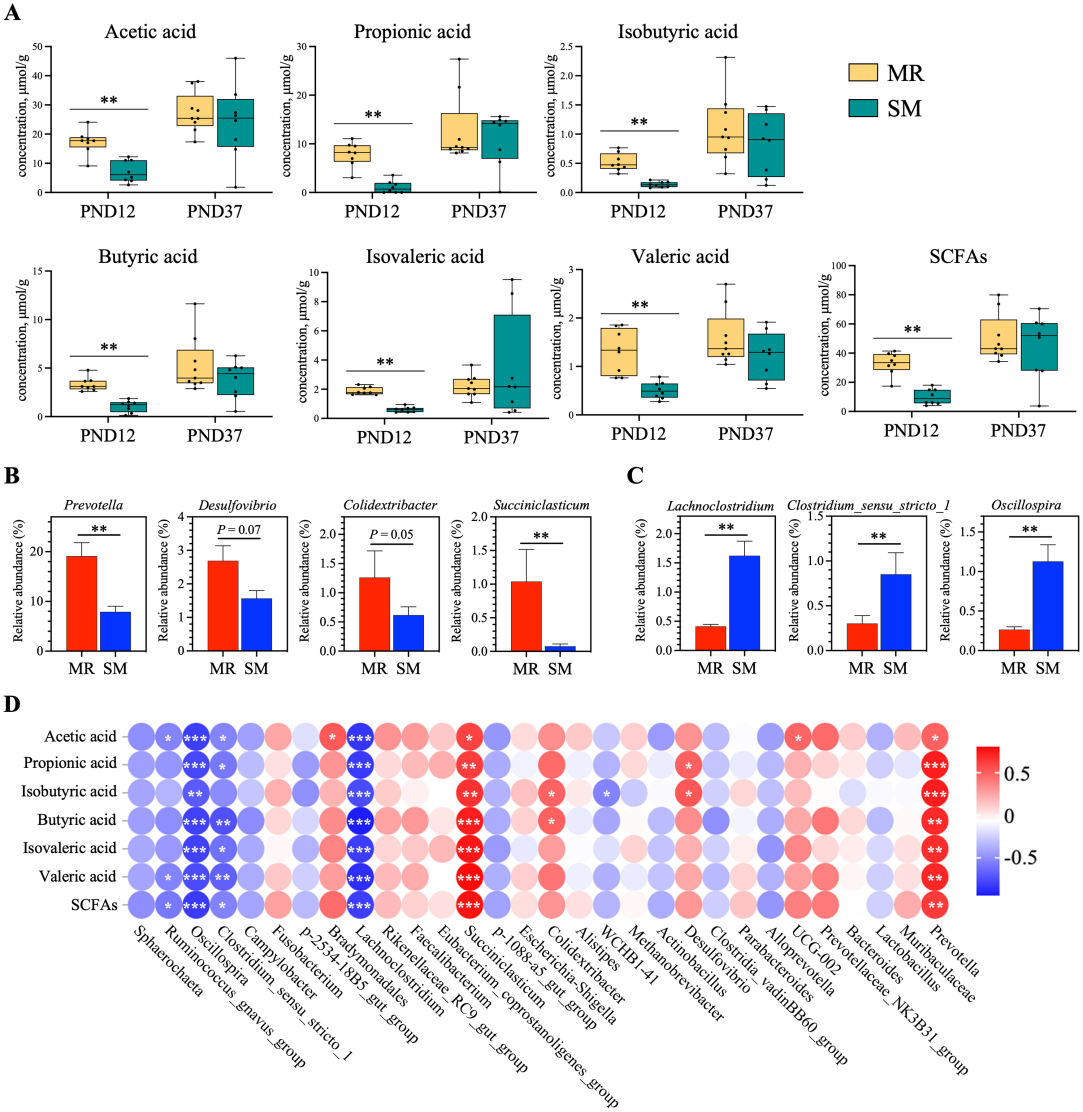
Effects of MR and SM on SCFAs concentration in piglets and correlation analysis. PND: postnatal day. **(A)** Concentrations of SCFAs in colonic digesta. **(B)** Bacteria significantly positively correlated with SCFAs were enriched in the MR group. **(C)** Bacteria significantly negatively correlated with SCFAs were enriched in the SM group. **(D)** Correlation analysis between bacteria and SCFAs on PND 12. Data are presented as mean ± SEM. The analysis is based on Spearman correlation coefficient. ^*^p < 0.05; ^**^p < 0.01; ^***^p < 0.001.

Compared to SM-fed piglets, MR-fed piglets had higher concentrations of acetic acid, propionic acid, isobutyric acid, butyric acid, isovaleric acid, valeric acid and SCFAs on PND 12 (*p* < 0.01, Figure 9A).

### 3.9 Correlation analysis

We conducted a correlation analysis between the top 30 bacteria and short-chain fatty acids (SCFAs) (Figure 9D) and performed statistical analysis on the genera with a correlation to SCFAs of *p* < 0.05 (Figures 9B,C). The results revealed that *Prevotella*, *Desulfovibrio*, *Colidextribacter* and *Succiniclasticum*, which had higher abundances in MR-fed piglets, exhibited a significant positive correlation with SCFAs. However, *Lachnoclostridium*, *Clostridium_sensu_stricto_1*, and *Oscillospira*, which had higher abundances in SM-fed piglets, showed a significant negative correlation with SCFAs.

## 4 Discussion

This study found that piglets receiving MR immediately after colostrum had a poorer growth rate than those fed SM, likely due to early separation stress and environmental changes. Specifically, MR-fed piglets showed approximately a 10% lower ADG during the neonatal period. Similar finding has been reported, where piglets fed MR from PND 3 to PND 10 had reduced average daily gain (−112 g/d) compared to SM-fed piglets (8). SM provides essential nutrients and bioactive compounds crucial for the growth and health of neonatal piglets, such as immunoglobulins, cytokines, and growth factors (30). These compounds are largely deficient in MR, which may be the main factors leading to poorer growth rates. Similar result has been reported that MR-fed piglets showed a lower growth rate (240 g/d vs. 270 g/d, *p* < 0.001) from 7 to 26 days of age (7). However, the growth restriction observed in MR-fed piglets was resolved during the nursery period. MR-fed piglets demonstrated 62.7% higher ADG and 48.5% higher ADFI compared to SM-fed piglets. The higher ADFI in MR-fed piglets during the nursery period may be attributed to their early adaptation to non-maternal feeding during neonatal period, which likely reduces weaning stress. Meanwhile, aligning with previous findings, MR-fed piglets could achieve catch-up growth as those suckling SM over a longer period (8, 31, 32).

The fat content of diet is crucial for neonatal piglets, as early fat deposition influences their growth and survival (33). In this study, SM or MR differentially affected piglet body composition. During the neonatal period, MR-fed piglets had lower body fat compared to SM-fed piglets, which is expected due to the higher fat content in SM relative to MR (10, 11). *Hansen* et al. previously analyzed SM composition across 27 studies and found that the fat content in SM (36.30% of DM) was higher than in MR (25.42% of DM) (34). This discrepancy in dietary fat content could be also reflected in the plasma lipid profiles of the piglets. MR-fed piglets showed significantly lower levels of total cholesterol (TC), low-density lipoprotein cholesterol (LDL-C), and high-density lipoprotein cholesterol (HDL-C), indicating lower lipid absorption and mobilization. TC and LDL-C are crucial for cellular membrane synthesis and steroid hormone production, while HDL-C facilitates reverse cholesterol transport (35, 36). The abnormal lipid markers in MR-fed piglets suggest compromised lipid utilization, likely due to insufficient dietary fat intake or an altered fatty acid composition in MR compared to SM (37). In addition, studies have shown that muscle fiber types are influenced by factors, such as age, breed and nutrition (15). This study found that early MR feeding negatively affected muscle fiber type conversion during the neonatal period, resulting in a significantly lower percentage of fast-twitch fibers (II). At birth, pig muscle fibers are primarily oxidative (I, IIa), and rapidly convert to glycolytic fibers (IIb, IIx) (13). Feeding MR during the neonatal period appears to limit this transformation. It should be noted that the transition from oxidative to glycolytic fibers is crucial for the muscular development and overall growth performance (38). The limited conversion in MR-fed piglets may be attributed to the nutrient composition between milk replacer and sow milk, particularly in terms of protein quality, amino acid profile, and bioactive compounds (39). However, this effect of MR on muscle fiber composition appeared to be transient. MR-fed piglets were able to recover fiber type conversion during nursery period, suggesting that muscle development during early life stages is plastic (40). This plasticity may serve as an adaptive mechanism, allowing piglets to adjust to different nutritional environments and ensuring optimal muscle development. Despite this recovery, MR-fed piglets still had lower body fat compared to SM-fed piglets, which may be related to differences in energy intake or nutrient utilization between the two group. Nevertheless, the underlying mechanisms through which MR effects body composition require further investigation.

Early separation from the dam subjects neonatal piglets to psychological and environmental stress, impairing small intestinal morphology (18), increasing intestinal inflammation (41, 42), and disrupting colonic microbiota composition (43). In this study, dietary changes and separation stress likely contributed to the lower growth performance and higher diarrhea rates in MR-fed piglets during the neonatal period. Along with higher diarrhea, supportively, MR-fed piglets showed deeper crypt, increased relative mRNA expressions of inflammatory genes, and changes in microbial composition during the neonatal period. Crypt hyperplasia indicates impaired intestinal function (44), and deeper crypts were observed in MR-fed neonatal piglets at 10 and 28 days of age (8). Similar findings have been reported in studies of human infants and rat pups (45, 46). The intestine is the largest immune organ in animals, with many immune cells gathered in the intestinal mucosa (47). Stress from separating piglets from their sows, along with dietary and environmental changes, can induce transient intestinal inflammation in piglets, characterized by increased pro-inflammatory cytokines *TNF-α*, *IL-6*, and *IL-1β* (48). As expected, the relative mRNA expressions of inflammation-related genes like *TNF-α* and *IL-6* were upregulated in the ileum of MR-fed neonatal piglets. However, this inflammation typically resolved within 2 weeks during the nursery period, as complex adaptive responses occurred in MR-fed piglets, as evidenced by higher expressions of tight junction genes (*Claudin-1*, *Occludin*, *ZO-1*) and innate immunity-related genes (*TLR-4*, *TRAF-6*, *NF-κB*, *MyD88*). The TLR family, crucial for intestinal mucosal defense and homeostasis (49), along with its downstream signaling molecules, participates in immune responses through multiple pathways (50, 51). Notably, the TLR-4–MyD88–NF-κB signaling pathway is implicated in inflammatory responses (50). These results suggest that milk replacer feeding may induce temporary intestinal damage and microbial dysbiosis, while simultaneously activating compensatory mechanisms in the piglet’s immune and barrier systems. These adaptive responses likely help piglets cope with the transition stress.

The composition of intestinal microbiota is crucial for pig growth and health and is significantly affected by diet (52, 53). In this study, MR-fed piglets exhibited lower alpha diversity of microbiota during the neonatal period. PCoA analysis also revealed differences in beta diversity between the MR and SM groups, suggesting that dietary sources altered the intestinal microbiota composition. To minimize the impact of environmental microbes, sow feces were collected every 2 days and smeared in the MR piglet crates, aiming to provide a microbial environment similar to that of sow-reared piglets. Although this method may not fully replicate the microbial composition, studies suggest environmental microbial exposure influences gut microbiota development and immune responses in piglets (54, 55). Changes in the functional gene profile of microbiota suggest that the transient imbalance may create a favorable environment for the rapid proliferation of *Prevotella* (56), the dominant bacteria found in MR-fed piglets with diarrhea. *Lachnospiraceae* has been shown to be enriched in the intestines of obese humans (57, 58). Similarly, this study found that *Lachnospiraceae* was enriched in the intestines of SM-fed piglets with greater fat deposition. Additionally, our findings reveal a complex relationship between feeding methods, gut microbiota, and SCFA production in neonatal piglets. The higher SCFA concentrations might be explained by the enrichment of SCFA-producing bacteria like *Prevotella* and *Desulfovibrio* in the MR group (59). Despite of better overall performance, lower SCFA levels in SM piglets could be attributed to the higher abundances of bacteria negatively correlated with SCFAs, such as *Lachnoclostridium* and *Clostridium_sensu_stricto_1* (60). Likewise, the potentially impaired intestinal function in nursery piglets appeared to recover, as evidenced by a lower diarrhea index, higher villus height, increased sucrase activity, and improved microbiota composition. An increase in villus height indicates a greater intestinal surface area and enhanced nutrient absorption (61). The higher sucrase activity in the jejunum of MR-fed piglets benefits carbohydrate digestion. A similar disaccharidase maturation pattern was reported in a previous study, suggesting that MR-fed piglets partially adapted to the weaning diet (8). In this study, MR-fed piglets showed lower alpha diversity of microbiota during the neonatal period but showed higher diversity during the nursery period, indicating that the initial decrease in microbial alpha diversity gradually recovered over time (62, 63). Beneficial bacteria such as f_Muribaculaceae, *g_Prevotellaceae_NK3B31_group*, f_Oscillospiraceae and f_Rikenellaceae were enriched in the intestines of MR-fed piglets (64–66). The enrichment of *Prevotellaceae_NK3B31_group* in MR-fed piglets during the nursery period may facilitate the transition from milk to solid diet, as this group is typically enriched in the healthy intestines of healthy weaning piglets (66). These results indicate that the higher growth rate in MR-fed piglets during the nursery period may be due to better adaptation to environmental changes, improved intestinal function, and a more beneficial microbiota.

## 5 Conclusion

The replacement of MR with SM may lead to temporary growth check and intestinal complications during the neonatal period, but intriguingly MR-fed piglets had higher feed intake, compensatory growth, and recovery of intestinal function during the nursery period. This findings suggested that early separation from the dam and environmental changes stimulate physiological adaptation to subsequent transition stresses, including dietary and environmental shifts, from the neonatal to the nursery period. Future studies should focus on the long-term effects of early dam separation and MR feeding on growth, intestinal and muscular development, as well as the underlying mechanisms involved.

## Data Availability

The raw sequencing data generated for this study have been deposited in the NCBI Sequence Read Archive (SRA) under accession number [PRJNA1232341]. The data supporting the findings are included in the article. For further inquiries, contact the corresponding author.
